# Glucagon‐like peptide‐1 receptor agonists for neurological disorders: a systematic review and meta‐analysis

**DOI:** 10.1002/alz70859_098720

**Published:** 2025-12-25

**Authors:** Apostolos Manolopoulos, Konstantinos Malandris, Josephine M. Egan, Dimitrios Kapogiannis

**Affiliations:** ^1^ National Institute on Aging, Baltimore, MD USA; ^2^ Aristotle University of Thessaloniki, Thessaloniki, Central Macedonia Greece

## Abstract

**Background:**

Glucagon‐like peptide‐1 receptor agonists (GLP‐1RAs) are currently approved for type 2 diabetes mellitus, obesity, and obstructive sleep apnea. However, due to the presence of GLP‐1 receptors on certain neuronal populations in the brain, GLP‐1RAs are being investigated for their therapeutic potential in a variety of neurological conditions. We conducted a systematic review and meta‐analysis to evaluate the efficacy of GLP‐1RAs in neurological disorders.

**Method:**

We searched MEDLINE, Embase, Cochrane Library, and ClinicalTrials.gov from inception to 4 August 2024 for randomized controlled trials (RCTs) in adults with Alzheimer’s disease (AD), Parkinson’s disease (PD), ischemic stroke, idiopathic intracranial hypertension (IIH), or hypothalamic obesity (HO) comparing GLP‐1RAs to placebo. Primary outcomes included a composite of memory and cognition for AD, MDS‐UPDRS Part III (ON and OFF states) for PD, NIHSS for stroke, intracranial pressure for IIH, and body mass index (BMI) for HO. Data synthesis was conducted using Review Manager, with risk of bias assessed via the revised Cochrane Risk of Bias tool and evidence certainty evaluated using the GRADE approach.

**Result:**

Fifteen RCTs involving 1341 participants were included. In AD, GLP‐1RAs did not improve memory and cognition compared to placebo (standardized mean difference [SMD] ‐0.01, 95% confidence intervals [CIs] ‐0.44 to 0.41; low‐certainty evidence). In PD, no improvement was observed in MDS‐UPDRS Part III (OFF state); however, improvement was noted in the ON state (mean difference [MD] ‐2.15, 95% CIs ‐3.46 to ‐0.83; moderate‐certainty evidence). In HO, GLP‐1RAs reduced BMI (MD ‐0.75, 95% CIs ‐1.35 to ‐0.15; moderate‐certainty evidence). No significant differences were observed for NIHSS in stroke or intracranial pressure in IIH.

**Conclusion:**

Low‐to‐moderate certainty evidence indicates that GLP‐1RAs provide modest benefits in specific conditions, including motor function improvement in PD and BMI reduction in HO, without however any evident effects in improving cognition in AD, recovery after stroke, or reducing intracranial pressure in IIH. Future high‐quality trials are warranted to identify optimal treatment strategies, understand patient‐specific responses, and further explore the mechanisms of action of GLP‐1RAs in neurological conditions.

